# Plaster cast versus functional brace for non-surgical treatment of Achilles tendon rupture (UKSTAR): a multicentre randomised controlled trial and economic evaluation

**DOI:** 10.1016/S0140-6736(19)32942-3

**Published:** 2020-02-08

**Authors:** Matthew L Costa, Juul Achten, Ioana R Marian, Susan J Dutton, Sarah E Lamb, Benjamin Ollivere, Mandy Maredza, Stavros Petrou, Rebecca S Kearney, UKSTAR trial collaborators, Amr Abdallah, Moez Ballal, Jordi Ballester, James Beastall, Rajarshi Bhattacharya, Simon Burrt, Mark Deakin, Rupe Deol, Asterios Dramis, Sameh El-Kawy, Jason Eyre, Justin Forder, Avijeet Ghosh, Anhijit Guha, Nicholas Hancock, Fraser Harrold, Paul Harwood, Peter Hull, Alan Johnstone, Sandeep Kapoor, Babis Karagkevrekis, Andrew Kelly, Nasser Kurdy, Harish Kurup, Victoria Lyle, Sanjeev Madan, Jane Madeley, Ansar Mahmood, Atif Malik, Andrew McAndrew, Viren Mishra, Nitin Modi, Rajesh Nanda, Ines Reichert, Nikos Reissis, Sridhar Sampalli, Andrea Scott, Richard Walter, Mark Westwood

**Affiliations:** a Oxford Trauma, Nuffield Department of Rheumatology, Musculoskeletal and Orthopaedic Sciences, University of Oxford, Oxford, UK; b Oxford Clinical Trials Research Unit, Centre for Statistics in Medicine, Nuffield Department of Rheumatology, Musculoskeletal and Orthopaedic Sciences, University of Oxford, Oxford, UK; c College of Medicine and Health, University of Exeter, Exeter, UK; d Division of Rheumatology, Orthopaedics and Dermatology, School of Medicine, University of Nottingham, Nottingham, UK; e Warwick Clinical Trials Unit, University of Warwick, Coventry, UK; f Nuffield Department of Primary Care Health Sciences, University of Oxford, Oxford, UK

## Abstract

**Background:**

Patients with Achilles tendon rupture who have non-operative treatment have traditionally been treated with immobilisation of the tendon in plaster casts for several weeks. Functional bracing is an alternative non-operative treatment that allows earlier mobilisation, but evidence on its effectiveness and safety is scarce. The aim of the UKSTAR trial was to compare functional and quality-of-life outcomes and resource use in patients treated non-operatively with plaster cast versus functional brace.

**Methods:**

UKSTAR was a pragmatic, superiority, multicentre, randomised controlled trial done at 39 hospitals in the UK. Patients (aged ≥16 years) who were being treated non-operatively for a primary Achilles tendon rupture at the participating centres were potentially eligible. The exclusion criteria were presenting more than 14 days after injury, previous rupture of the same Achilles tendon, or being unable to complete the questionnaires. Eligible participants were randomly assigned (1:1) to receive a plaster cast or functional brace using a centralised web-based system. Because the interventions were clearly visible, neither patients nor clinicians could be masked. Participants wore the intervention for 8 weeks. The primary outcome was patient-reported Achilles tendon rupture score (ATRS) at 9 months, analysed in the modified intention-to-treat population (all patients in the groups to which they were allocated, excluding participants who withdrew or died before providing any outcome data). The main safety outcome was the incidence of tendon re-rupture. Resource use was recorded from a health and personal social care perspective. The trial is registered with ISRCTN, ISRCTN62639639.

**Findings:**

Between Aug 15, 2016, and May 31, 2018, 1451 patients were screened, of whom 540 participants (mean age 48·7 years, 79% male) were randomly allocated to receive plaster cast (n=266) or functional brace (n=274). 527 (98%) of 540 were included in the modified intention-to-treat population, and 13 (2%) were excluded because they withdrew or died before providing any outcome data. There was no difference in ATRS at 9 months post injury (cast group n=244, mean ATRS 74∙4 [SD 19∙8]; functional brace group n=259, ATRS 72∙8 [20∙4]; adjusted mean difference –1∙38 [95% CI –4∙9 to 2∙1], p=0·44). There was no difference in the rate of re-rupture of the tendon (17 [6%] of 266 in the plaster cast group *vs* 13 [5%] of 274 in the functional brace group, p=0·40). The mean total health and personal social care cost was £1181 for the plaster cast group and £1078 for the functional bract group (mean between-group difference –£103 [95% CI –289 to 84]).

**Interpretation:**

Traditional plaster casting was not found to be superior to early weight-bearing in a functional brace, as measured by ATRS, in the management of patients treated non-surgically for Achilles tendon rupture. Clinicians may consider the use of early weight-bearing in a functional brace as a safe and cost-effective alternative to plaster casting.

**Funding:**

UK National Institute for Health Research Health Technology Assessment Programme.

## Introduction

Rupture of the Achilles tendon is an increasingly common injury in both the sporting and non-sporting populations, leading to a prolonged period away from work and social activities.^1^ The most recent trials comparing surgical repair with non-operative treatment have found no difference in functional outcome; therefore, non-operative treatment is increasingly preferred by clinicians and patients.^2^, ^3^, ^4^

Traditionally, patients with an Achilles rupture have been treated with serial plaster casts over several weeks. The cast provides maximum protection for the tendon as it heals, but immobilisation might increase calf muscle atrophy, ankle joint stiffness, gait abnormalities, and the risk of blood clots.^5^, ^6^, ^7^ Functional bracing is an alternative treatment in which the patient's lower leg is placed into a removable walking boot which contains wedges to lift up the heel. The brace allows the patient to put weight through their leg as they walk and can be removed to allow movement at the ankle joint. However, evidence is scarce on how functional bracing affects overall recovery and whether it is associated with an increased risk of re-rupture of the tendon.^8^

**Research in context**

**Evidence before this study**

In the past 10 years, trials comparing surgical repair with non​-surgical treatment for patients with a rupture of the Achilles tendon have found no evidence of a difference in functional outcome; therefore, non-surgical treatment is increasingly preferred. However, before this study, little evidence was available on the best type of non-surgical treatment. Traditionally, patients have been treated in plaster casts to immobilise the foot and ankle while the tendon heals. However, this approach has an immediate effect on mobility for a period of around eight weeks, affecting activities of daily life. Prolonged immobilisation is associated with risk of muscle atrophy, deep vein thrombosis, and joint stiffness. In addition, it has potential long-term consequences, including prolonged gait abnormalities, persistent calf muscle weakness, and an inability to return to previous activity levels. Functional bracing, involving immediate, protected weight-bearing in a brace, was designed to address these issues. However, before the UKSTAR trial, little evidence regarding its effectiveness was available, and some clinicians had concerns about safety, in particular the risk of re-rupture of the tendon. In guidelines published in 2009, the American Academy of Orthopaedic Surgeons (AAOS) stated that it was unable to recommend for or against the use of functional bracing for patients with Achilles tendon rupture treated non-operatively. We updated this evidence synthesis in 2019 by doing a literature search of MEDLINE and Embase with the terms “Achilles tendon” AND “rupture” for articles published in English from 2009 (date of the AAOS guidelines) to October, 2019. To our knowledge, the only trials published to date have been single-centre studies with small patient numbers and inconclusive findings.

**Added value of this study**

In the UKSTAR trial, 540 adult participants at 39 hospitals in the UK were randomly assigned to either plaster cast or functional brace for non-surgical treatment of an Achilles tendon rupture. The study found no evidence that traditional plaster casting is superior to early weight-bearing in a functional brace as measured by Achilles tendon rupture score. The results also showed no evidence of a difference in the rate of re-rupture of the tendon. A health economic analysis indicated that functional bracing is likely to be cost-effective.

**Implications of all the available evidence**

Rupture of the Achilles tendon is a serious and increasingly common injury. In keeping with the latest evidence, patients are increasingly choosing to have their tendon treated non-surgically. However, to date, little evidence was available on the best type of non-surgical treatment. The findings of the UKSTAR trial will assist patients and clinicians in choosing the most suitable non-surgical treatment. Furthermore, policy makers will note that the health economic evaluation indicates that functional bracing is likely to be cost-effective.

The objective of the UKSTAR trial was to compare function and pain, quality of life, complications (including re-rupture), and resource use in patients having non-operative treatment for an acute Achilles tendon rupture, treated with plaster cast versus functional bracing.

## Methods

### Study design

UKSTAR was a pragmatic, superiority, multicentre, randomised controlled trial done at 39 National Health Service (NHS) hospitals in the UK. The study was given a favourable Research Ethics opinion by the Oxford B Research Ethics Committee on April 7, 2016 (reference 16/SC/0109) and each recruitment centre was granted site-specific approval from its NHS Trust Research and Development department before trial commencement. The detailed protocol and statistical analysis plan have been published previously.^9^, ^10^

### Participants

All patients aged 16 years or older who presented at the trial centres with a primary (first-time) rupture of the Achilles tendon were screened. The patient, in conjunction with their surgeon, decided whether non-surgical treatment was appropriate, as per normal clinical practice. If patients decided not to have surgery, they were potentially eligible to take part in the trial. Patients were excluded if they presented more than 14 days after their injury, had experienced a previous rupture of the same Achilles tendon, or were unable to complete questionnaires. Eligible patients were provided with the trial information verbally and in writing. When the patient had considered the trial information, informed written consent was obtained by a trained member of the research team.

### Randomisation and masking

Participants were randomly allocated (1:1) to either functional bracing or plaster cast using a computer-generated allocation sequence stratified by recruitment centre via a secure, centralised web-based randomisation service. The sequence was prepared by the trial statistician. Stratification by recruitment centre helped to ensure any cluster effect related to the recruitment centre itself was equally distributed in the trial groups. A local research associate informed the treating clinical team of the allocated treatment. Because the interventions were clearly visible, neither patients nor clinicians could be masked.

### Procedures

The interventions were chosen on the basis of a systematic review of the literature and a survey of current UK clinical practice; plaster casting is the most common treatment, and rigid braces are used more commonly than flexible orthotics.^4^, ^8^ Images of the two treatments are provided in the appendix (p 2).

Participants who were randomly allocated to the plaster cast group had a below-knee plaster cast applied in the gravity equinus position (ie, the position that the foot naturally adopts when unsupported, with the toes pointing down towards the floor). Over the first 8 weeks, the participants returned to the hospital (the frequency of visits was decided by the clinical team at that hospital)and the position of the plaster cast was gradually changed until the foot was in a plantigrade position (ie, the foot was flat to the floor). At this point, usually 6 weeks after the first cast was applied, the patient was permitted to start fully weight-bearing in the plaster cast. The cast was removed at 8 weeks.

Participants who were randomly allocated to the functional brace group had a removable, rigid walking boot. Initially, two solid heel wedges (or equivalent) were inserted inside the brace to replicate the gravity equinus position of the foot.^11^ However, because the bottom of the brace was flat to the floor, the participant was able to mobilise with their full weight on their foot immediately after fitting. The brace also permitted some movement at the ankle joint. The number of wedges and foot position were reduced over 8 weeks until the foot reached a plantigrade position. The brace was removed at 8 weeks.

At 8 weeks, all participants were provided with the same standardised, written physiotherapy advice detailing the exercises they needed to perform for rehabilitation (appendix p 1). This standardised rehabilitation advice was based on a published systematic review of rehabilitation protocols and refined through a previous feasibility trial.^8^ All participants were advised to move their toes, ankle joint, and knee joint fully within the limits of their comfort, and walking was encouraged. In this pragmatic trial, any other rehabilitation input beyond the written physiotherapy advice (including a formal referral to physiotherapy) was recorded but left to the discretion of the treating clinicians, as per their normal clinical practice. Patients were assessed for outcomes at 8 weeks and followed up at 3, 6, and 9 months from randomisation. All primary and secondary outcomes were assessed at all visits.

### Outcomes

The primary outcome was Achilles tendon rupture score (ATRS) at 9 months.^12^ ATRS is patient-reported and consists of 10 items that assess symptoms, physical activity, and pain related to the Achilles tendon to give a score between 0 and 100, with 100 as the best possible score.

The secondary outcomes were ATRS at other timepoints, health-related quality of life (measured by EQ-5D-5L),^13^ and complications (tendon re-rupture, deep vein thrombosis, pulmonary embolism, fall with no injury, fall with injury, pain under the heel, numbness around the foot, and pressure sores).

### Statistical and health economic analysis

The minimum clinically important difference (MCID) for the primary outcome ATRS was 8 points. At an individual patient level, a difference of 8 points represents the ability to walk upstairs or run with some difficulty versus with great difficulty. At the population level, 8 points represents the difference between a healthy patient and a patient with a minor disability.^14^ In our pilot work, the standard deviation of the ATRS at 9 months post injury was 20 points.^15^ Assuming a likely population variability of 20, MCID value of 8, 90% power to detect the selected MCID, and a 5% type 1 error rate on a two-sided test, we required 264 total participants to be randomised. Allowing a margin of 20% loss of primary outcome data to include patients who would cross over between interventions or be lost to follow-up, we required a minimum of 330 participants. Recruitment was faster than anticipated, and, with the approval of the Research Ethics Committee, we were able to exceed the minimum number of participants required in the sample size calculation to obtain a more precise estimate of the number of complications.

The primary outcome of ATRS at 9 months was analysed in a modified intention-to-treat population. We also considered the complier average causal effect (CACE) population to account for compliance with treatment.^16^ The modified intention-to-treat population included all participants in their randomised groups, excluding participants who had missing data at baseline, or who withdrew or died before providing any outcome data. The CACE population included all randomised participants who were compliant with treatment. Participants were considered compliant with the intervention if they wore their allocated treatment for a period of 6 weeks or more without any change of treatment within this period. We used a linear mixed effects regression model, adjusting for age, gender, and baseline ATRS as fixed effects, and recruitment centre and repeated measures within participants as random effects. Secondary outcomes were analysed in the modified intention-to-treat population, with use of similar methods adjusting for the relevant baseline covariate if applicable. Complications were analysed using χ^2^ or Fisher's exact test in the intention-to-treat population. Sensitivity analyses to examine the robustness of conclusions to different assumptions were done in the CACE population. All analyses were done using Stata version 15.0 (StataCorp, College Station, TX, USA).

The health economic evaluation adopted an NHS and personal social services perspective, in accordance with UK National Institute for Health and Care Excellence recommendations.^17^ A societal perspective for costs was adopted for the sensitivity analysis and this included private costs incurred by trial participants and their families, and productivity losses and loss of earnings as a result of work absences. The economic evaluation was a cost-utility analysis, expressed in terms of incremental cost per quality-adjusted life-year (QALY) gained. The time horizon covered the period from randomisation to end of follow-up at 9 months post injury. Economic costs associated with the direct delivery of the interventions were estimated and included costs of the walking boot and wedges, materials used for plaster casts, and the costs associated with fitting the interventions to patients (hospital staff time). Resource-use questionnaires completed by participants at each follow-up timepoint provided a profile of broader NHS and personal social services resource use. Resource-use values were converted into costs (in GBP, 2017–18 prices) by applying unit costs obtained from key UK national databases.^18^, ^19^, ^20^ Further details of costing procedures are provided in the appendix (pp 3–5). QALY profiles were calculated for each participant using health utility scores generated from the EQ-5D-5L and assuming linear interpolation between baseline and follow-up health utility scores. Bivariate regression of costs and QALYs, with multiple imputation for missing data, was conducted in order to estimate the incremental cost per QALY gained for functional bracing compared with plaster cast. Furthermore, the probability of cost-effectiveness of functional bracing was estimated over a range of cost-effectiveness thresholds: £15 000, £20 000, and £30 000 per QALY gained.^17^, ^21^ Further details on the methods of the economic evaluation and the sensitivity analyses done to test the robustness of cost-effectiveness results are provided in the appendix (pp 5, 6).

The trial is registered with ISRCTN, ISRCTN62639639.

### Role of the funding source

The funders of the study had no role in study design, data collection, data analysis, data interpretation, or writing of the report. The corresponding author had full access to all the data in the study and had final responsibility for the decision to submit for publication.

## Results

Between Aug 15, 2016, and May 31, 2018**,** 1451 patients were screened, of whom 540 participants were randomly allocated to receive plaster cast (n=266) or functional brace (n=274; figure). One participant in each group withdrew consent immediately pre-treatment. Participants had a mean age of 48∙7 years (SD 13·8), were predominantly male (426 [79%] of 538), and most commonly ruptured their tendon during sports (70%). The study groups were well balanced on all baseline characteristics (table 1). 13 participants were excluded from the modified intention-to-treat population, because they had missing data at baseline (n=4), or withdrew (n=8) or died (n=1) before providing any outcome data. 527 (98%) participants were included in the primary outcome analysis.

**Figure:**
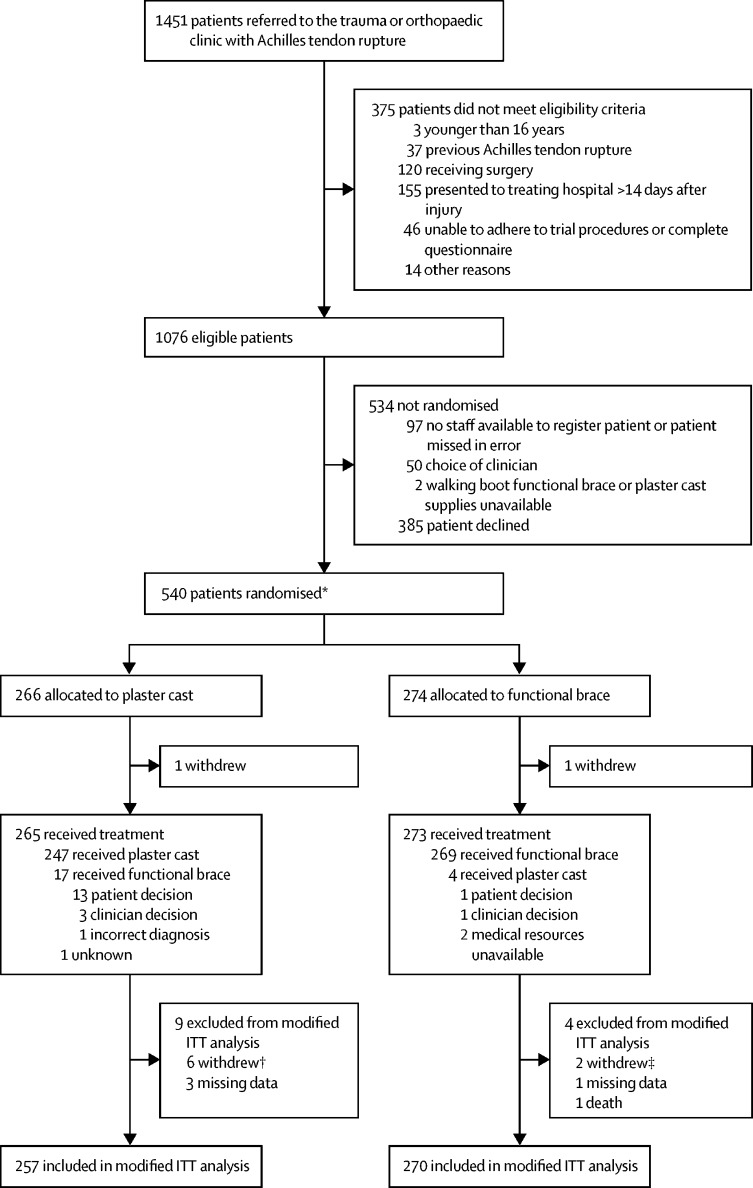
Trial profile ATRS=Achilles tendon rupture score. ITT=intention-to-treat. *Two participants were randomised in error without giving consent to be in the study, and they immediately withdrew as shown; these participants were excluded from all data analyses. † Includes one patient who withdrew pre-treatment and five patients who withdrew post-treatment. ‡ Includes one patient who withdrew pre-treatment and one patient with withdrew post-treatment.

**Table 1:** Baseline characteristics

		**Plaster cast (n=264)**	**Functional brace (n=274)**	**Overall (n=538)**
Gender				
	Female	51 (19%)	61 (22%)	112 (21%)
	Male	213 (81%)	213 (78%)	426 (79%)
Age, years		49·0 (14·0)	48·3 (13·8)	48·7 (13·8)
ATRS pre-injury				
	n	264	273	537
	Median (IQR)	100·0 (96·5–100·0)	100·0 (94·0–100·0)	100·0 (96·0–100·0)
BMI, kg/m^2^				
	n	255	265	520
	Mean (SD)	27·5 (4·5)	27·8 (5·0)	27·7 (4·8)
Days since injury		5·0 (2·5–8·0)	5·0 (2·0–8·0)	5·0 (2·0–8·0)
Mechanism of injury				
	Fall from height	3 (1%)	8 (3%)	11 (2%)
	Fall on steps or stairs	22 (8%)	14 (5%)	36 (7%)
	Fall or trip from standing height	6 (2%)	11 (4%)	17 (3%)
	Pushing an object	18 (7%)	15 (5%)	33 (6%)
	Sports	187 (71%)	192 (70%)	379 (70%)
	Walking	14 (5%)	28 (10%)	42 (8%)
	Other	14 (5%)	6 (2%)	20 (4%)
Side of injury				
	Right	122 (46%)	138 (50%)	260 (48%)
	Left	142 (54%)	136 (50%)	278 (52%)
Regular smoker				
	No	225 (85%)	234 (85%)	459 (85%)
	Yes	39 (15%)	39 (14%)	78 (14%)
	Missing	0	1 (<1%)	1 (<1%)
Cigarettes per day				
	n	39	39	78
	Median (IQR)	10 (5–15)	10 (5–15)	10 (5–15)
Smoking duration, years				
	n	38	38	76
	Median (IQR)	20·0 (10·0–25·0)	20·5 (13·0–30·0)	20·0 (10·0–30·0)
Alcohol units per week				
	0–7	162 (61%)	161 (59%)	323 (60%)
	8–14	49 (19%)	65 (24%)	114 (21%)
	15–21	40 (15%)	35 (13%)	75 (14%)
	>21	12 (5%)	10 (4%)	22 (4%)
	Missing	1 (<1%)	3 (1%)	4 (1%)
Taking medication				
	Fluoroquinolone antibiotics	5 (2%)	4 (2%)	9 (2%)
	Steroids	7 (3%)	14 (5%)	21 (4%)
	DMARDs	2 (1%)	1 (<1%)	3 (1%)
	Diabetic medication	5 (2%)	14 (5%)	19 (4%)
	Regular analgesia	23 (9%)	14 (5%)	37 (7%)
	Anticoagulant medication	66 (25%)	78 (29%)	144 (27%)
Diagnosis before injury				
	Diabetes	5 (2%)	18 (7%)	23 (4%)
	Rheumatoid arthritis	0	3 (1%)	3 (1%)
	Lower limb fracture (past 12 months)	1 (<1%)	4 (1%)	5 (1%)
	Ligament, tendon, or nerve injury to lower limb (past 12 months)	5 (2%)	8 (3%)	13 (2%)
	Arthritis	21 (8%)	21 (8%)	42 (8%)
	Achilles tendinopathy	10 (4%)	10 (4%)	20 (4%)
Employment status				
	Full-time employed	160 (61%)	168 (61%)	328 (61%)
	Part-time employed	18 (7%)	15 (5%)	33 (6%)
	Self-employed	39 (15%)	29 (11%)	68 (13%)
	Retired, looking after home, or inactive	35 (13%)	41 (15%)	76 (14%)
	Unpaid work	1 (<1%)	2 (1%)	3 (1%)
	Unemployed	8 (3%)	8 (3%)	16 (3%)
	Full-time student	3 (1%)	9 (3%)	12 (2%)
	Missing	0	2 (1%)	2 (<1%)
Employment category				
	Unskilled manual	11 (4%)	11 (4%)	22 (4%)
	Skilled manual	62 (23%)	64 (23%)	126 (23%)
	Unskilled non-manual	6 (2%)	7 (3%)	13 (2%)
	Skilled non-manual	29 (11%)	21 (8%)	50 (9%)
	Professional	109 (41%)	108 (39%)	217 (40%)
	Missing	0	3 (1%)	3 (1%)

Data are n (%) for categorical variables and mean (SD) or median (IQR) for continuous outcomes unless stated otherwise. ATRS=Achilles tendon rupture score. BMI=body-mass index. DMARD=disease-modifying antirheumatic drug.

There was no statistically or clinically significant difference in ATRS at 9 months post injury in the plaster cast group (mean ATRS 74∙4 [SD 19∙8]) versus the functional brace group (72∙8 [20∙4]; adjusted mean difference –1∙38 [95% CI –4∙9 to 2∙1]; table 2). There was no statistically significant difference in the secondary CACE analysis (adjusted mean difference –1∙18 (–4∙5 to 2∙1). We found a statistically significant but clinically equivocal difference in ATRS at 8 weeks post injury in favour of functional brace (adjusted mean difference 5∙53 [2∙0–9∙1]), but not at 3 months or 6 months post injury. Health-related quality of life, measured by EQ-5D-5L, showed the same pattern, with a statistically significant difference at 8 weeks post injury but not at later timepoints (appendix p 15). There was no evidence of a difference in the complication profiles between groups (table 3). 17 (6%) of 266 participants in the plaster cast group had re-rupture of the tendon, compared with 13 (5%) in the functional brace group.

**Table 2:** ATRS in the modified intention-to-treat population

	**Plaster cast**		**Functional brace**		**Between-group difference (95% CI)**		**p value**
	n	ATRS, mean (SD)	n	ATRS, mean (SD)	Unadjusted	Adjusted*	
8 weeks	234	35·3 (20·1)	240	40·3 (17·8)	4·98 (1·3 to 8·7)	5·53 (2·0 to 9·1)	0·0024
3 months	229	44·4 (21·1)	244	45·6 (20·4)	1·23 (−2·5 to 4·9)	1·76 (−1·8 to 5·3)	0·34
6 months	224	63·9 (21·4)	235	63·5 (23·0)	−0·44 (−4·2 to 3·3)	0·35 (−3·3 to 4·0)	0·85
9 months	244	74·4 (19·8)	259	72·8 (20·4)	−1·65 (−5·2 to 1·9)	−1·38 (−4·9 to 2·1)	0·44

The analysis was based on a mixed effects model with repeated measures from all timepoints. ATRS=Achilles tendon rupture score.

* ATRS analysis adjusted for recruitment centre, age, gender, and baseline ATRS with repeated observations within participant.

**Table 3:** Complications from baseline to 9 months in the intention-to-treat population

	**Plaster cast (n=266)**	**Functional brace (n=274)**	**p value**
Tendon re-rupture	17 (6%)	13 (5%)	0·40
Deep vein thrombosis	3 (1%)	6 (2%)	0·51
Pulmonary embolism	0	2 (1%)	0·50
Fall with no injury	60 (23%)	53 (19%)	0·36
Fall with injury sustained	21 (8%)	24 (9%)	0·72
Pain under the heel	158 (59%)	180 (66%)	0·13
Numbness around the foot	108 (41%)	130 (47%)	0·11
Pressure sores	39 (15%)	51 (19%)	0·22

Data are number of participants reporting complication at least once (%). Complications in each treatment group were summed over the 9-month follow-up.

The mean direct intervention costs were £36 for the plaster cast group compared with £109 for the functional brace group (mean difference £73 [95% CI 67–79]; p<0·0001). The mean total NHS and personal social services cost throughout the entire follow-up period for the base case (imputed) analysis was £1181 for the plaster cast group and £1078 for the functional brace group. Although functional bracing was slightly cheaper, the mean between-group cost difference of –£103 (95% CI –289 to 84) was not statistically significant. The QALY estimate for the base case was slightly higher for patients in the functional brace group (0∙015 QALYs [95% CI –0∙001 to 0∙030]) over the 9-month follow-up period. The incremental cost-effectiveness ratio for the base-case analysis showed that functional bracing was the dominant procedure because the functional brace group incurred slightly lower costs and experienced slightly higher QALYs over the follow-up period. The probability of cost-effectiveness for functional bracing was 0·97 at a cost-effectiveness threshold of £20 000 per QALY gained. This finding remained robust to most sensitivity analyses; the exception was an assessment of cost-effectiveness from a societal perspective in which the probability of cost-effectiveness for functional bracing was 0∙58. Further results of the economic evaluation are provided in the appendix (pp 7–20).

## Discussion

The findings of this study showed no difference in ATRS at 9 months between plaster cast and functional bracing for patients treated non-operatively for a rupture of the Achilles tendon. There was a statistically significant difference in ATRS at 8 weeks in favour of the functional brace group, although the clinical significance of this difference is debatable. Any benefit to functional bracing at 8 weeks was not evident later in recovery, with similar scores between groups at 3, 6, and 9 months. Health-related quality of life showed a similar pattern of recovery over time. The difference in EQ-5D-5L utility score was significant early in the patients’ recovery, but there was no evidence of a difference at 9 months.

The safety profile of the functional brace was another important consideration of this trial. Specifically, if the risk of re-rupture of the tendon were higher in patients who were allowed to fully weight-bear in a functional brace, this would influence the decision to choose this treatment, even if patient-reported outcomes were similar. We found that the risk of re-rupture was generally lower than that reported in the literature,^22^ with 17 (6%) patients experiencing re-rupture in the plaster cast group and 13 (5%) in the functional brace group. None of the re-ruptures occurred more than 6 months after the injury.

When UKSTAR was developed, the scarcity of evidence in this area was recognised in the 2009 American Academy of Orthopaedic Surgeons guideline,^23^ which concluded that “For patients treated non-operatively, we are unable to recommend for or against the use of immediate functional bracing for patients with acute Achilles tendon rupture.” Since the start of the UKSTAR trial, a number of small randomised trials have investigated both the mechanistic and functional effects of early weight-bearing in a brace versus cast immobilisation for patients treated non-operatively. One trial investigated the biomechanical properties of the healing tendon in patients randomly allocated to early weight-bearing versus delayed weight-bearing.^24^ The investigators noted that the group treated with early weight-bearing experienced less tendon stiffness. However, in terms of functional outcomes, the authors reported no evidence of a difference.^25^ A second trial of 47 participants investigated weight-bearing in patients treated non-operatively for an acute Achilles tendon rupture. Half of the patients were treated with partial weight-bearing beginning on the first day of treatment and the other half with non-weight-bearing for the first 4 weeks. The authors concluded that early weight-bearing was safe, in terms of the incidence of re-rupture, but they found no evidence of a difference in functional outcome in the first 12 months after the rupture.^26^ Another trial of 84 patients compared two types of cast immobilisation of the Achilles tendon rupture.^27^ Half of the patients wore a traditional cast, which restricted weight-bearing, and the other group wore a modified cast which included a heel iron to facilitate weight-bearing. The authors found no evidence of a difference in functional outcome. One further study, published in 2019,^28^ randomly allocated 130 patients to cast immobilisation or early controlled motion at a single centre. The authors found no evidence that early controlled motion was of benefit compared with immobilisation in any of the investigated outcomes.

The strengths of the pragmatic UKSTAR trial were the use of multiple centres and clinicians reflecting the care provided across the UK NHS, the large number of participants with 93% complete follow-up, and the use of validated patient-reported outcomes.

As in all clinical trials, some patients declined to participate. In this trial, we approached only individuals who had already decided against surgical repair. The proportion of people accepting the invitation to participate was high, with most of those declining doing so because of an unwillingness to participate in research. Therefore, we believe the results to be generalisable to the population of patients having non-operative treatment for an acute rupture of the Achilles tendon. A further anticipated limitation was crossover from the allocated trial treatment. However, most participants received their allocated treatment with only 21 patients changing their allocated intervention after being randomised. In addition, some patients had incomplete compliance with treatment. The ability to bear weight immediately within a functional brace might have motivated patients to change from plaster cast to functional brace, given that these were most of the crossovers, rather than the other way around. However, the overall number of patients not receiving their allocated treatment is small and the CACE analysis, which was adjusted for incomplete compliance, confirmed the result of the primary analysis showing no evidence of a difference in ATRS between the two groups. Loss to follow-up is another potential limitation. However, over 93% of participants provided primary outcome data at 9 months, which is considerably higher than the 80% assumed in the trial design. Therefore, given that the trial also exceeded the minimum sample size, we can be confident that the conclusions based on the primary outcome are robust and the risk of type II error is low.

In conclusion, this trial provides no evidence that traditional plaster casting is superior to early weight-bearing in a functional brace as measured by ATRS in the management of patients treated non-operatively for Achilles tendon rupture. The use of functional bracing is likely to be cost-effective.

## Data sharing

All data requests should be submitted to the corresponding author for consideration. Access to anonymised data may be granted following review.
