# Supplementary material for: Plaster cast versus functional brace for non-surgical treatment of Achilles tendon rupture (UKSTAR): a multicentre randomised controlled trial and economic evaluation
Source: Lancet. 2020 Feb 8;395(10222):441–8. doi: 10.1016/S0140-6736(19)32942-3 (PMC7016510; doi:10.1016/S0140-6736(19)32942-3)
Supplement: Supplementary appendix [file mmc1.pdf]

THE LANCET

Supplementary appendix

This appendix formed part of the original submission and has been peer reviewed. We post it as supplied by the authors.

Supplement to: Costa ML, Achten J, Marian IR, et al. Plaster cast versus functional brace for non-surgical treatment of Achilles tendon rupture (UKSTAR): a multicentre randomised controlled trial and economic evaluation. *Lancet* 2020; **395:** 441–48.

Appendix 1

Physiotherapy Advice	
**Weeks 8-12**	• Your physiotherapist will advise you on how to wean you off your crutches, if you are still using them, and how to improve your walking pattern.<br>• If your ankle is swollen they will advise you to elevate your leg and apply ice to the ankle.<br>• You will begin regular range of movement exercises throughout the day for the ankle (for example 3 x 10 repetitions/day) and gentle resistance exercises, using a theraband that the physiotherapist will show you.
**Weeks 12-24**	• You will begin specific strength training for your calf muscles. This will consist of doing exercises that are low impact and require you to complete multiple repetitions, within your capabilities. Examples include double leg heel raises progressing to single leg heel raises, as appropriate.<br>• Your physiotherapist will also introduce some generic strengthening and balance exercises to prepare you later on for more demanding activities, if required. Such exercises may include mini squats, cycling, balancing skills and step ups. Again all are examples of low impact exercises. This will be alongside additional low impact exercises that you may choose to undertake such as cycling, jogging, and swimming.<br>• At this stage your calf muscles may begin to feel stiff or tight. If this is the case the physiotherapists will show you some active calf stretching exercises that will help.<br>• If your ankle movements still remain reduced at this stage the physiotherapist may progress to manual mobilization of the ankle.
**Week 24 onwards**	• By 24 weeks you will have returned to completing your normal activities of daily living such as driving, walking and going up and down stairs, and you will be able to take part in low impact sports such as cycling, jogging and swimming.<br>• If you wish to return to higher impact sports that involve sprinting, lunging and jumping, such as football, squash or tennis, then your physiotherapist will guide you through some sport specific exercises prior to you returning fully to these sports. Once you are able to complete the sport specific exercises then the physiotherapist will advise that you can return to these sporting activities.

Note: The above physiotherapy advice is a guide only. Your physiotherapist in consultation with your doctor may decide it is in your best interests to add or remove the specific physiotherapy treatments you receive within the above time frames.

The UKSTAR trial is funded by the National Institute for Health Research's HTA Programme.

Appendix 2

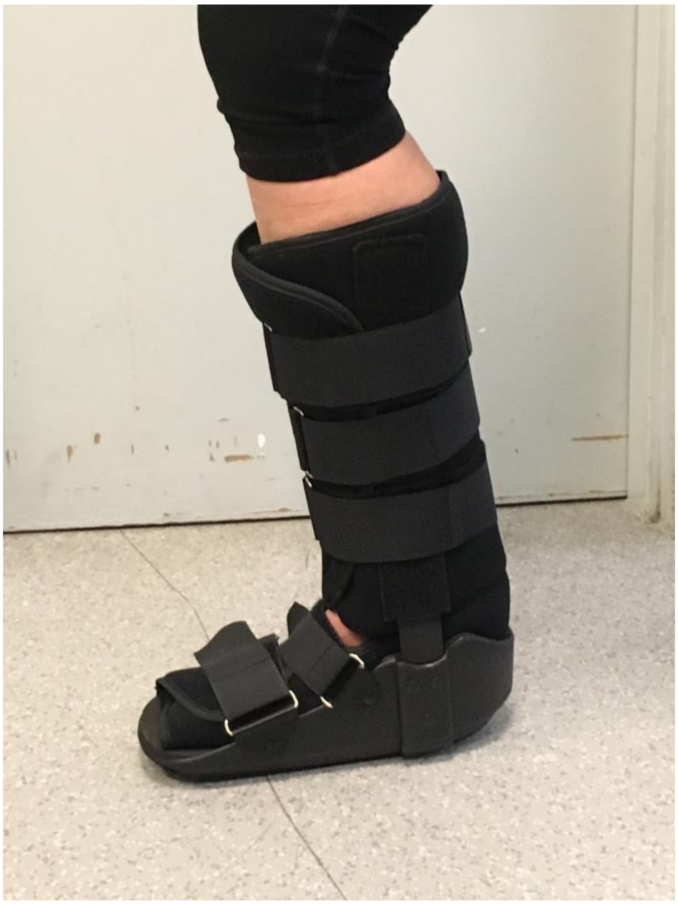

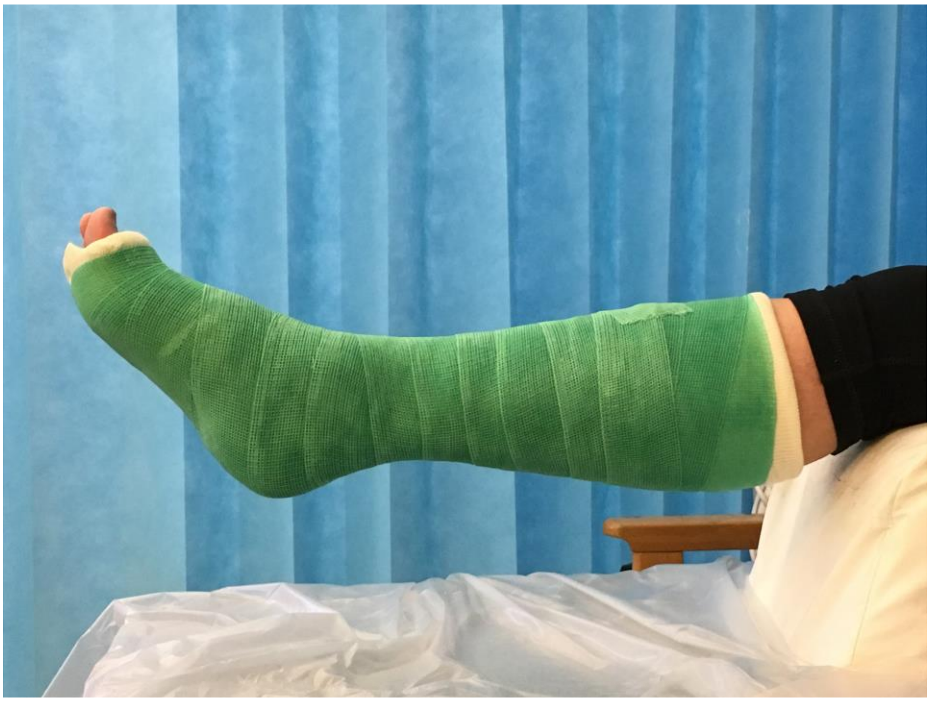

Appendix 3

UKSTAR: UK Study of Tendo Achilles Rehabilitation

Rehabilitation strategy after non-surgical treatment of Achilles tendon rupture: UKSTAR, a multicentre RCT

Appendix: Economic Evaluation

1. Overview

The objective of the health economic evaluation was to assess the comparative cost-effectiveness of the two non-surgical treatment options (plaster cast versus functional bracing) for patients with a primary (first-time) rupture of the Achilles tendon. To achieve this, a systematic comparison of the cost of resource inputs used by participants in the two arms of the trial and consequences associated with the interventions was conducted. The primary analysis adopted a National Health Service (NHS) and personal social services (PSS) perspective, in accordance with National Institute for Health and Care Excellence (NICE) recommendations [1]. A societal perspective for costs was adopted for the sensitivity analysis and this included private costs incurred by trial participants and their families, as well as productivity losses and loss of earnings as a result of work absences. The economic evaluation took the form of a cost–utility analysis, expressed in terms of incremental cost per quality adjusted life-year (QALY) gained. The time horizon covered the period from randomisation to end of follow-up at 9 months post injury. Costs and outcomes were not discounted due to the short, 9-month, time horizon adopted for this within-trial evaluation.

2. Measurement of resource use and costs

Data were collected on:

- i) Resource use and costs associated with delivery of the interventions (direct intervention costs)
- ii) Broader health and social care service use during the 9 months of follow-up
- iii) Broader societal resource use and costs – this encompassed private medical costs and lost productivity costs such as lost income over the 9 months of follow-up.

All costs were expressed in pounds sterling and valued in 2017-18 prices. When appropriate, costs were inflated or deflated to 2017–18 prices using the Hospital and Community Health Services (HCHS) Pay and Price Inflation Index [2].

2.1 Direct intervention costs

Direct intervention costs comprised costs associated with the application of the two interventions. This included cost of the walking boot and wedges, materials used for plaster cast, the cost associated with fitting the interventions to patients (hospital staff time), and the costs associated with any changes required to either plaster cast or functional bracing (Table A1). Information on how long it takes to deliver each intervention and type and volume of materials used was collected at each recruitment centre, through a questionnaire completed by recruitment centre staff in consultation with staff responsible for fitting the functional brace or applying the plaster cast. Unit costs for staff were obtained from the Personal Social Services Research Unit (PSSRU) Unit Costs of Health and Social Care 2018 compendium [3] and were multiplied by the median time it takes to deliver each intervention. The median time for fitting a functional brace was 10, 11 and 17·5 minutes for a plaster technician, nurse and other[1] staff respectively. The median time to change wedges was 5 minutes for a plaster technician and nurse and 10 minutes for ‘other’ staff. The median time for changing a plaster cast was 15 minutes for a plaster technician and 17·5 minutes for a nurse. The base case analysis assumed costs of a plaster technician. Unit costs of plaster cast materials, walking boots and wedges were obtained from the

[1] Other staff category included Physiotherapists, orthotists and occupational therapists

2018 NHS Supply chain catalogue. The total direct intervention cost for each patient was calculated by combining the resource inputs with their respective unit cost values.

Table A1: Unit costs associated with direct intervention costs for plaster cast and functional bracing

Resource item	Unit cost	Unit of analysis	Source of unit cost
**Direct intervention costs**			
**Functional brace:**			
Walking boot[2] cost by brand:			
Samson walking boot<br>Donjoy walking boot<br>Airstep walking boot	£15·00<br>£19·24<br>£68·66	Per walking boot	John Radcliffe finance department; NHS Supply Chain Catalogue 2018
**Plaster cast:**			
Plaster cast materials[3]			
2 x 7·5cm poly rolls<br>2x 10 cm poly rolls	£2·83<br>£6·69	Per roll	NHS Supply Chain Catalogue 2018
Fibreglass casting tape 5 inch x3·6m	£11·48	Per roll	NHS Supply Chain Catalogue 2018
1m stockinette	£3·23	Per roll	NHS Supply Chain Catalogue 2018
2 x rolls of 5inch wool bandage	£3·00	Per roll	NHS Supply Chain Catalogue 2018

2.2 Measuring broader resource use

Broader resource use data were collected using follow-up questionnaires completed by trial participants at the four follow-up assessment points: 8 weeks, 3 months, 6 months and 9 months post injury. The questionnaires captured details of inpatient and day case admissions, outpatient and emergency care attendances, encounters with primary or community health and social care services, medication use and walking aids provided/self-purchased, as well adaptations to home environments. In addition, the questionnaires captured the direct non-medical costs (including travel expenses) incurred by patients and their carers, as well as number of days off work and gross loss of earnings attributable to the trial participant's health state or contacts with care providers.

2.3 Valuation of resource use

Resource inputs were valued by attaching unit costs derived from national compendia in accordance with NICE's Guide to the Methods of Technology Appraisal 2013 [1]. The key databases for deriving unit cost data included the Department of Health and Social Care's Reference Costs 2016–17 schedules [4], the Personal Social Services Research Unit (PSSRU)'s Unit Costs of Health and Social Care 2018 compendium [3], the 2018 NHS Prescription Cost Analysis database for England [5], 2018 volumes of the British National Formulary [6], and the NHS Supply Chain Catalogue 2018 [7].

Per diem costs for hospital inpatient admissions during the follow-up period were calculated individually as a weighted average of Healthcare Resource Group (HRG) codes of related procedures and/or clinical diagnoses. For example, the average cost per day for an inpatient stay in a medical ward to treat a pulmonary embolus was calculated as the sum total of weighted average HRG codes (DZ09J – DZ09Q; pulmonary embolus with or without interventions) divided by average length of stay across elective and non-elective inpatient services. The individual HRG codes were derived using the NHS HRG4 2017/18 Reference Cost Grouper software version

[2] Unit costs for all other walking boot brands that patients received (not pre-specified in case report forms) were individually-derived from the NHS Supply Chain Catalogue

[3] Unit costs for any other plaster cast materials that sites use (not pre-specified in site-specific questionnaire) were individually-derived from the NHS Supply Chain Catalogue 2018

RC1718 (NHS Digital, Leeds, UK). The Department of Health and Social Care's *Reference Costs 2017–18 [4]* schedule was used to assign the costs for each of the derived HRG codes.

Costs for community-based health and social care services were calculated by applying unit costs extracted from national tariffs, primarily extracted from the PSSRU Unit Costs of Health and Social Care 2018 compendium [3], to resource volumes. Costs of medications for individual participants were estimated based on their reported doses and frequencies, when these were available, or based on an assumed daily doses using *British National Formulary* [6] recommendations. When a dose range was reported as 'as required' or when the quantities were not recorded, we assumed a mean cost for that medication item based on the prescription cost analysis values (net ingredient cost per item). In cases where medication dosages were missing, we conservatively assumed that the patient received the same dosage as other trial participants who reported taking the same medication.

The costs of walking aids and adaptations (equipment participants receive to manage their injury and make daily lives easier) were derived by combining data on number and type of items received with their unit cost values. Unit cost values were derived from the NHS supply chain catalogue [7] if equipment was provided by a health provider during the trial follow-up period. Where aids and adaptations were self-financed, the costs were provided by participants themselves.

We used data on sex and employment status-specific median earnings from the UK national annual survey of hours and earnings [8] to derive the costs of time taken off work. The employment status of trial participants was derived from self-reported work status information. Broader societal costs were calculated by combining the productivity losses and income losses attributable to work absences.

Summary statistics were generated for resource use variables by treatment allocation and assessment point. Between treatment-group differences in resource use and costs at each assessment point were compared using the two sample t-test. Statistical significance was assessed at the 5% significance level. Standard errors are reported for treatment group means and bootstrap 95% confidence intervals for the between-group differences in mean resource use and cost estimates.

3. Measurement of outcomes

In accordance with NICE guidelines, the primary health outcome for the health economic evaluation was the quality adjusted life year (QALY) metric [1]. Health-related quality of life of trial participants was assessed at baseline (both pre and post injury), and 8 weeks, 3 months, 6 months and 9 months post injury using the EuroQoL EQ-5D-5L instrument [9]. The EQ-5D-5L instrument defines health-related quality of life in terms of five dimensions: (1) mobility, (2) self-care, (3) usual activities, (4) pain/discomfort and (5) anxiety/depression. Responses in each dimension are divided into five ordinal levels coded: (1) no problems, (2) slight problems, (3) moderate problems, (4) severe problems, and (5) extreme problems. Between-group differences in optimal versus sub-optimal level of function for each health dimension were compared at each time-point using chi-squared ($\chi 2$) tests.

Responses to the EQ-5D-5L instrument were converted into health utility scores using the EQ-5D-5L Crosswalk Index Value Calculator currently recommended by NICE [10], which maps the EQ-5D-5L descriptive system data onto the EQ-5D-3L valuation set. Detailed description on the mapping methodology is described elsewhere [10]. Quality-adjusted life-years (QALYs) were generated for each patient using the area under the baseline-adjusted utility curve, assuming linear interpolation between health utility measurements across assessment points.

Health utility values and QALYs accrued over the 9-month follow-up period were summarised by treatment group and assessment point and presented as means and associated standard errors; between group differences were compared using the two-sample t-test, similar to the descriptive analyses of resource inputs and costs.

4. Cost-effectiveness analysis methods

4.1 Missing data

Missing data are a common occurrence within randomised controlled trials: participants may be lost to follow-up, questionnaires unreturned or responses to individual questionnaire items may be missing [11]. Because costs and outcomes of individuals with missing data may differ systematically to those with fully observed

data, it is important to handle missing data using a principled approach that is justified by, amongst other factors, the missing data mechanism. Missing costs and health utility data were imputed at each time-point using fully conditional multiple imputation by chain equations, implemented through the MICE package, under the missing at random (MAR) assumption. Appropriateness of the MAR assumption was assessed by: (i) investigating the missing data patterns (monotonic vs. non-monotonic), and (ii) comparing attributes of participants with and without missing costs and health-related quality of life data at each follow-up time-point. The multiple imputation model used baseline covariates (age, gender), costs and health utility values at each follow-up time-point to impute unobserved costs and health utility values, such that, for example, missing costs at 9 months were imputed using data on baseline covariates, costs at 8 week, 3 months and 6 months and health utility values at each follow-up time-point. The imputations were implemented separately by treatment allocation in line with best practice [11]. The imputation was run 50 times, following the rule of thumb that the number of imputations should be at least greater than the proportion of missing data [11]. Bivariate regressions using a seemingly unrelated regression model (Sureg) were used to independently analyse the multiply imputed datasets so as to estimate the costs and QALYs in each treatment group over the 9-month trial horizon. Joint distributions of costs and outcomes from the original data set were generated through non-parametric bootstrapping and changes in costs and QALYs were calculated for each sample. A total of 1000 bootstrap samples were drawn and means for both incremental costs and incremental QALYs (with associated 95% CIs) were calculated. Estimates from each imputed dataset were combined using Rubin's rule [12] to generate overall mean estimates of costs and QALYs and their standard errors (SE). The latter reflects the variability within and across imputations. The imputation model was validated by assessing the distributions of imputed and observed values. A mixed model with adjustment for baseline pre-injury EQ-5D health utility scores is also presented for comparison.

4.2 Presentation of cost-effectiveness results

Cost-effectiveness results are expressed in terms of the incremental cost-effectiveness ratio (ICER) and calculated as the difference between treatments in mean total costs divided by mean total QALYs. Given the pattern of results, plaster cast has been selected as the referent and functional brace as the comparator, i.e. functional brace minus plaster cast, for the estimation of ICER values. The bootstrap replicates generated by the non-parametric bootstrapping, described in the sub-section 'Missing data', were used to populate cost-effectiveness scatterplots. Cost-effectiveness acceptability curves, which showed the probability that functional brace is cost-effective relative to plaster cast across a range of cost-effectiveness thresholds, were also generated based on the proportion of bootstrap replicates with positive incremental net benefits. The net monetary benefit (NMB) of using functional brace versus plaster cast was also calculated across three pre-specified cost-effectiveness thresholds, namely £15,000 per QALY [13], £20,000 per QALY and £30,000 per QALY [1]. A positive incremental NMB indicates that the functional brace is cost-effective compared with the plaster cast at the given cost-effectiveness threshold. For the purpose of the secondary analysis that adopted the ATRS as the health outcome measure of interest, the NMB was estimated at cost-effectiveness thresholds of £100 - £500 per unit change in ATRS score. We failed to identify any external evidence on economic values for changes in ATRS score and therefore a range of arbitrary threshold values had to be selected for this analysis.

4.3 Sensitivity and secondary outcomes analyses

Several sensitivity analyses were conducted to test the robustness of the cost-effectiveness estimates. These involved re-estimating the main cost-effectiveness outcomes under the following scenarios: (1) restricting the analyses to complete cases (i.e. those with complete cost and outcome data over the 9-month follow-up period); (2) adopting a wider societal perspective that included private costs incurred by trial participants and their families, as well as economic losses placed on attributable work absences; and (3) Estimating incremental cost-effectiveness using a CACE population. In addition, as a secondary analysis, cost-effectiveness was estimated using the ATRS, rather than the QALY, as the health outcome measure of interest.

4.4 Longer-term economic modelling

The study protocol also allowed for decision-analytic modelling to estimate longer-term cost-effectiveness of functional bracing or plaster cast provided the costs and health outcomes did not converge at the end of the 9-month post injury follow-up period.

5. Results of economic analysis

Table A2 shows the degree of missing health economic data by treatment allocation and follow-up time point. The missing data pattern is non-monotonic, as individuals with missing data at one follow-up time point may return to the trial subsequently. For example, there are more missing EQ-5D data at 6 months than at 9 months post injury. A similar pattern can be observed for economic costs. It is worth noting that the lower number of participants with complete data for the entire duration of follow-up (baseline to 9 months post-injury) was due to a strict application of the term missing i.e. we considered a participant as having incomplete data if, for example, they responded positively to vising a GP surgery at 3 months but did not specify number of consultations, despite all other resource use items being completed. However, for the cost-effectiveness analysis, imputation was not done at the aggregate level such that most of the data used for the analysis was based on actual participant responses.

Table A2: Number and proportion of individuals with missing health economic data by treatment allocation

Variable	Description	Treatment group, missing values, *n* (%)		Total, missing values, *n (%)*
		**Plaster cast (n=266)**	**Functional brace (n = 274)**	
eq5db	EQ-5D index score pre injury	2 (0·75)	2 (0·73)	4 (0·74)
eq5d0	EQ-5D index score post injury	2 (0·75)	1 (0·36)	3 (0·56)
eq5d1	EQ-5D at 8 weeks	32 (12·06)	33 (12·04)	65 (12·04)
eq5d2	EQ-5D at 3 months	37 (13·91)	29 (10·58)	66 (12·22)
eq5d3	EQ-5D at 6 months	42 (15·79)	37 (13·5)	79 (14·63)
eq5d4	EQ-5D at 9 months	22 (26)	15 (5·47)	37 (8·27)
QALY	QALYs generated from EQ-5D utility scores	76 (28·57)	74 (27·01)	149 (27·78)
c0	Total resource use between baseline and 8 weeks post injury	66 (24·8)	59 (21·53)	125 (23·15)
c1	Total resource use between 8 weeks and 3 months post injury	59 (22·18)	47 (17·15)	106 (19·63)
c2	Total resource use between 3 and 6 months post injury	56 (21·05)	48 (8·89)	104 (19·26)
c3	Total resource use between 6 and 9 months post injury	31 (11·65)	18 (6·57)	49 (9·07)
c4	Total resource use between baseline and 9 months post injury	132 (49·62)	116 (42·34)	248 (45·93)

QALY: quality adjusted life-year

5.1 Health and social care resource use

In terms of specific resource use for plaster cast versus functional brace for all participants at the 8 week follow-up, notable differences were observed for: proportion prescribed anticoagulant as VTE prophylaxis treatment (0·72 vs 0·59; p=0·003), mean number of NHS outpatient orthopaedic visits (2·63 vs 1·80; p<0·001), mean number of NHS outpatient physiotherapy visits (0·23 vs 0·46; p=0·003), mean number of GP surgery visits (0·10 vs 0·19; p=0·028), and mean number of grab rail installations (0·05 vs 0; p=0·019). For all other resource use items, there were no noticeable differences between the trial groups.

Between 8 week and 3 months post injury, for all participants there were differences in resource use for plaster cast versus functional brace observed for: proportion of participants prescribed analgesics (0·11 vs 0·05; p=0·015) and proportion of participants prescribed other medications (0·02 vs 0; p=0·038). For all other resource use items, there were no noticeable differences between the trial groups.

There were no significant differences in resource use for the Plaster Cast versus Functional Brace trial groups at 6 months and 9 months post injury. Table A3 provides details on use of health care resources by follow-up time-point and treatment group.

Table A3: Use of health and social care resources related to two non-surgical treatment options for patients with a primary (first-time) rupture of the Achilles tendon by each follow-up period and treatment grop (complete cases)

	Plaster Cast (N=266)	Functional brace (N=274)	Mean difference (Bootstrapped 95% CI)
**8-week follow-up**			
**Inpatient Care**	**Mean length of stay in days (SE)**		
Hospital stay	0·035 (0·018)	0·071 (0·032)	-0·036 (-0·110 to 0·028)
	**Proportion of participants prescribed anticoagulant as VTE prophylaxis treatment (SE)**		
**Anticoagulant treatment**	0·716 (0·028)	0·594 (0·030)	0·122 (0·032 to 0·200)
Outpatient care	**Mean no· of visits (SE)**		
Orthopaedics	2·627 (0·107)	1·800 (0·097)	0·827 (0·574 to 1·119)
Pathology	0·041 (0·014)	0·068 (0·023)	-0·027 (-0·084 to 0·025)
Radiology	0·150 (0·023)	0·146 (0·029)	0·004 (-0·070 to 0·074)
Physiotherapy NHS	0·228 (0·042)	0·460 (0·064)	-0·232 (-0·397 to -0·095)
Physiotherapy Private	0·091 (0·037)	0·184 (0·160)	-0·093 (-0·575 to 0·117)
Emergency Department (Injury –related)	0·104 (0·023)	0·096 (0·021)	0·008 (-0·051 to 0·070)
Emergency Department (other reasons)	0·029 (0·012)	0·016 (0·008)	0·013 (-0·012 to 0·044)
Other	0·111 (0·037)	0·168 (0·045)	-0·058 (-0·162 to 0·062)
Community health care	**Mean no· of contacts (SE)**		
GP Visits (surgery)	0·100 (0·024)	0·188 (0·032)	-0·088 (-0·176 to -0·012)
GP (home visits)	0·008 (0·006)	0 (0)	0·008 (0 to 0·024)
GP (telephone contacts)	0·084 (0·025)	0·108 (0·031)	-0·024 (-0·103 to 0·049)
Practice nurse contacts	0·008 (0·006)	0·008 (0·006)	0 (-0·015 to 0·018)
District nurse contacts	0·151 (0·146)	0 (0)	0·151 (0 to 0·553)
Community physiotherapy contacts	0·021 (0·013)	0·040 (0·020)	-0·019 (-0·074 to 0·020)
Calls to NHS direct	0·017 (0·010)	0·008 (0·008)	0·009 (-0·012 to 0·039)
Calls for an ambulance or paramedic	0·004 (0·004)	0 (0)	0·004 (0 to 0·017)
Occupational therapy contacts	0·013 (0·009)	0·008 (0·006)	0·005 (-0·012 to 0·034)
Other	0·216 (0·146)	0·034 (0·015)	0·183 (-0·010 to 0·580)
Medicines	**Proportion of participants prescribed each class of drug (SE)**		
Analgesics	0·388 (0·055)	0·330 (0·050)	0·058 (-0·083 to 0·213)
Anti-inflammatories	0·042 (0·013)	0·076 (0·017)	-0·034 (-0·081 to 0·004)
Anti-coagulant	0·151 (0·023)	0·112 (0·020)	0·039 (-0·026 to 0·093)
Other	0·017 (0·008)	0·048 (0·014)	-0·031 (-0·064 to -0·001)
Aids and adaptations	**Mean count (SE)**		
Crutches	1·290 (0·059)	1·124 (0·062)	0·166 (0·012 to 0·341)

	Plaster Cast (N=266)	Functional brace (N=274)	Mean difference (Bootstrapped 95% CI)
Stick	0·017 (0·010)	0·024 (0·010)	-0·007 (-0·033 to 0·024)
Zimmer frame	0·054 (0·018)	0·028 (0·010)	0·026 (-0·014 to 0·068)
Grab Rail	0·046 (0·020)	0 (0)	0·046 (0·013 to 0·090)
Dressing aids	0·008 (0·008)	0·008 (0·006)	0 (-0·016 to 0·024)
Long-handle shoe horn	0·004 (0·004)	0 (0)	0·004 (0 to 0·016)
Other	0·387 (0·045)	0·220 (0·043)	0·166 (0·040 to 0·277)
Personal social services	**No· of contacts (SE)**		
Frozen meals on wheels	0	0	-
Hot meals on wheels	0	0	-
Laundry services	0·029 (0·029)	0 (0)	0·029 (0 to 0·095)
Social worker contacts	0	0	-
Care worker/home help	0·668 (0·542)	0 (0)	0·668 (0 to 2·165)
Other	0	0	-
Productivity losses	**Mean days off work (SE)**		
Days off work	21·227 (1·682)	20·786 (1·637)	0·441 (-3·947 to 5·176)
Three-month follow-up			
**Inpatient Care**	**Mean length of stay in days (SE)**		
Hospital stay	0·009 (0·009)	0 (0)	0·009 (0 to 0·034)
Outpatient care	**Mean no· of visits (SE)**		
Orthopaedics	0·428 (0·055)	0·318 (0·045)	0·110 (-0·035 to 0·256)
Pathology	0·017 (0·011)	0·024 (0·014)	-0·007 (-0·044 to 0·026)
Radiology	0·057 (0·020)	0·045 (0·017)	0·012 (-0·038 to 0·062)
Physiotherapy NHS	0·978 (0·070)	0·959 (0·067)	0·019 (-0·175 to 0·180)
Physiotherapy Private	0·271 (0·073)	0·180 (0·045)	0·091 (-0·069 to 0·279)
Emergency Department (Injury –related)	0·061 (0·022)	0·033 (0·011)	0·028 (-0·013 to 0·085)
Emergency Department (other reasons)	0·009 (0·006)	0·004 (0·004)	0·005 (-0·008 to 0·023)
Other	0·057 (0·018)	0·050 (0·024)	0·007 (-0·057 to 0·062)
Community health care	**Mean no· of contacts (SE)**		
GP Visits (surgery)	0·088 (0·022)	0·107 (0·029)	-0·019 (-0·099 to 0·057)
GP (home visits)	0 (0)	(0)	-
GP (telephone contacts)	0·044 (0·017)	0·029 (0·013)	0·015 (-0·026 to 0·057)
Practice nurse contacts	0·004 (0·004)	0·008 (0·008)	-0·004 (-0·026 to 0·009)
District nurse contacts	0·004 (0·004)	0·004 (0·004)	0 (-0·011 to 0·013)
Community physiotherapy contacts	0·253 (0·065)	0·201 (0·044)	0·052 (-0·085 to 0·223)
Calls to NHS direct	0·004 (0·004)	0·004 (0·004)	0 (-0·009 to 0·013)
Calls for an ambulance or paramedic	0·013 (0·010)	0·004 (0·004)	0·009 (-0·008 to 0·036)
Occupational therapy contacts	0·022 (0·014)	0·049 (0·027)	-0·027 (-0·096 to 0·022)
Other	0·061 (0·032)	0·021 (0·012)	0·041 (-0·017 to 0·122)

	Plaster Cast (N=266)	Functional brace (N=274)	Mean difference (Bootstrapped 95% CI)
Medicines	**Proportion of participants prescribed each class of drug (SE)**		
Analgesics	0·109 (0·021)	0·049 (0·014)	0·060 (0·014 to 0·111)
Anti-inflammatories	0·008 (0·006)	0·008 (0·006)	0·001 (-0·015 to 0·019)
Anti-coagulant	0·022 (0·010)	0·016 (0·008)	0·005 (-0·019 to 0·031)
Other	0·017 (0·009)	0 (0)	0·017 (0·004 to 0·039)
Aids and adaptations	**Mean count (SE)**		
Crutches	0·118 (0·030)	0·106 (0·029)	0·012 (-0·071 to 0·100)
Stick	0·070 (0·20)	0·033 (0·014)	0·037 (-0·009 to 0·086)
Zimmer frame	0 (0)	0·004 (0·004)	0·004 (-0·016 to 0)
Grab Rail	0·022 (0·013)	0 (0)	0·022 (0 to 0·055)
Dressing aids	0·031 (0·020)	0 (0)	0·031 (0·004 to 0·083)
Long-handle shoe horn	0·013 (0·008)	0 (0)	0·013 (0 to 0·032)
Other	0·227 (0·064)	0·155 (0·038)	0·072 (-0·056 to 0·244)
Personal social services (PSS)	**No· of contacts (SE)**		
Frozen meals on wheels	0 (0)	0 (0)	-
Hot meals on wheels	0 (0)	0 (0)	-
Laundry services	0 (0)	0·008 (0·008)	-0·008 (-0·033 to 0)
Social worker contacts	0 (0)	0 (0)	-
Care worker/home help	0 (0)	0 (0)	-
Other	0·009 (0·009)	0 (0)	0·009 (0 to 0·029)
Productivity losses	**No· of days off work**		
Days off work	4·511 (0·820)	5·44 (0·880)	-0·930 (-3·342 to 1·494)
Six-month follow-up			
**Subsequent Inpatient Care**	**Mean length of stay in days (SE)**		
Hospital stay	0 (0)	0 (0)	-
Outpatient care	**Mean no· of visits (SE)**		
Orthopaedics	0·224 (0·043)	0·289 (0·059)	-0·065 (-0·230 to 0·071)
Pathology	0·018 (0·011)	0·030 (0·015)	-0·012 (-0·048 to 0·024)
Radiology	0·044 (0·015)	0·033 (0·012)	0·011 (-0·027 to 0·050)
Physiotherapy NHS	1·946 (0·257)	1·915 (0·182)	0·031 (-0·550 to 0·674)
Physiotherapy Private	0·417 (0·103)	0·366 (0·091)	0·051 (-0·218 to 0·316)
Emergency Department (Injury –related)	0·013 (0·008)	0·026 (0·010)	-0·012 (-0·039 to 0·014)
Emergency Department (other reasons)	0·013 (0·008)	0·017 (0·008)	-0·004 (-0·026 to 0·018)
Other	0·093 (0·031)	0·067 (0·031)	0·026 (-0·065 to 0·103)
Community health care	**Mean no· of contacts (SE)**		

	Plaster Cast (N=266)	Functional brace (N=274)	Mean difference (Bootstrapped 95% CI)
GP Visits (surgery)	0·094 (0·037)	0·060 (0·018)	0·035 (-0·034 to 0·122)
GP (home visits)	0 (0)	0·009 (0·009)	-0·009 (-0·028 to 0)
GP (telephone contacts)	0·018 (0·011)	0·021 (0·015)	-0·003 (-0·046 to 0·031)
Practice nurse contacts	0 (0)	0·004 (0·004)	-0·004 (-0·017 to 0)
District nurse contacts	0 (0)	0 (0)	-
Community physiotherapy contacts	0·605 (0·187)	0·557 (0·101)	0·048 (-0·311 to 0·547)
Calls to NHS direct	0 (0)	0 (0)	-
Calls for an ambulance or paramedic	0 (0)	0 (0)	-
Occupational therapy contacts	0·067 (0·033)	0·043 (0·023)	0·025 (-0·054 to 0·115)
Other	0·058 (0·043)	0·106 (0·077)	-0·048 (-0·264 to 0·085)
Medicines	**Proportion of participants prescribed each class of drug (SE)**		
Analgesics	0·103 (0·020)	0·064 (0·016)	0·040 (-0·008 to 0·090)
Anti-inflammatories	0·009 (0·006)	0·021 (0·009)	-0·012 (-0·036 to 0·009)
Anti-coagulant	0·004 (0·004)	0·013 (0·007)	-0·008 (-0·025 to 0·009)
Other	0·009 (0·006)	0·008 (0·006)	0 (-0·013 to 0·019)
Aids and adaptations	**Mean count (SE)**		
Crutches	0·054 (0·021)	0·051 (0·020)	0·003 (-0·051 to 0·066)
Stick	0·031 (0·012)	0·030 (0·015)	0·002 (-0·041 to 0·035)
Zimmer frame	0·009 (0·009)	0·013 (0·007)	-0·004 (-0·022)
Grab Rail	0·018 (0·011)	0·008 (0·008)	0·009 (-0·013 to 0·038)
Dressing aids	0 (0)	0 (0)	-
Long-handle shoe horn	0·018 (0·009)	0·008 (0·006)	0·009 (-0·009 to 0·032)
Other	0·144 (0·047)	0·091 (0·030)	0·054 (-0·031 to 0·188)
Personal social services	**No· of contacts (SE)**		
Frozen meals on wheels	0 (0)	0 (0)	-
Hot meals on wheels	0 (0)	0 (0)	-
Laundry services	0 (0)	0 (0)	-
Social worker contacts	0 (0)	0 (0)	-
Care worker/home help	0·036 (0·036)	0 (0)	0·036 (0 to 0·138)
Other	0 (0)	0 (0)	-
Productivity losses	**No· of days off work**		
Days off work	1·894 (0·743)	4·301 (1·172)	-2·407 (-5·642 to -0·110)
Nine-month follow-up			
**Subsequent Inpatient Care**	**Mean length of stay in days (SE)**		
Hospital stay	0 (0)	0 (0)	-

	Plaster Cast (N=266)	Functional brace (N=274)	Mean difference (Bootstrapped 95% CI)
Outpatient care	**Mean no· of visits (SE)**		
Orthopaedics	0·090 (0·024)	0·077 (0·030)	0·013 (-0·060 to 0·081)
Pathology	0·016 (0·008)	0·073 (0·027)	-0·057 (-0·120 to -0·012)
Radiology	0·029 (0·011)	0·012 (0·009)	0·017 (-0·005 to 0·047)
Physiotherapy NHS	0·709 (0·108)	0·857 (0·147)	-0·148 (-0·540 to 0·178)
Physiotherapy Private	0·234 (0·073)	0·174 (0·058)	0·060 (-0·103 to 0·260)
Emergency Department (injury –related)	0·004 (0·004)	0·008 (0·005)	-0·004 (-0·016 to 0·012)
Emergency Department (other reasons)	0·020 (0·011)	0·030 (0·014)	-0·010 (-0·051 to 0·019)
Other	0·140 (0·058)	0·089 (0·045)	0·051 (-0·090 to 0·206)
Community health care	**Mean no· of contacts (SE)**		
GP Visits (surgery)	0·058 (0·024)	0·046 (0·017)	0·011 (-0·046 to 0·072)
GP (home visits)	0 (0)	0 (0)	-
GP (telephone contacts)	0·008 (0·006)	0·004 (0·004)	0·004 (-0·007 to 0·021)
Practice nurse contacts	0 (0)	0·004 (0·004)	-0·004 (-0·016 to 0)
District nurse contacts	0 (0)	0 (0)	-
Community physiotherapy contacts	0·169 (0·052)	0·255 (0·066)	-0·085 (-0·258 to 0·071)
Calls to NHS direct	0 (0)	0 (0)	-
Calls for an ambulance or paramedic	0 (0)	0 (0)	-
Occupational therapy contacts	0·074 (0·038)	0·031 (0·017)	0·043 (-0·033 to 0·128)
Other	0·136 (0·070)	0·131 (0·100)	0·005 (-0·307 to 0·214)
Medicines	**Proportion of participants prescribed each class of drug (SE)**		
Analgesics	0·037 (0·012)	0·031 (0·011)	0·006 (-0·027 to 0·037)
Anti-inflammatories	0·012 (0·007)	0 (0)	0·012 (0 to 0·029)
Anti-coagulant	0·004 (0·004)	0 (0)	0·004 (0 to 0·016)
Other	0·004 (0·004)	0·004 (0·004)	0 (-0·008 to 0·016)
Aids and adaptations	**Mean count (SE)**		
Crutches	0 (0)	0·008 (0·008)	-0·008 (-0·029 to 0)
Stick	0·012 (0·009)	0·004 (0·004)	0·009 (-0·008 to 0·036)
Zimmer frame	0 (0)	0 (0)	-
Grab Rail	0·008 (0·008)	0 (0)	0·008 (0 to 0·031)
Dressing aids	0 (0)	0 (0)	-
Long-handle shoe horn	0·004 (0·004)	0 (0)	0·004 (0 to 0·017)
Other	0·062 (0·025)	0·093 (0·026)	-0·031 (-0·097 to 0·046)
Personal social services	**No· of contacts (SE)**		
Frozen meals on wheels	0·045 (0·045)	0 (0)	0·045 (0 to 0·182)
Hot meals on wheels	0 (0)	0 (0)	-
Laundry services	0·045 (0·045)	0 (0)	0·045 (0 to 0·182)

	Plaster Cast (N=266)	Functional brace (N=274)	Mean difference (Bootstrapped 95% CI)
Social worker contacts	0 (0)	0 (0)	-
Care worker/home help	0·008 (0·008)	0 (0)	0·008 (0 to 0·037)
Other	0 (0)	0 (0)	-
Productivity losses	**No· of days off work**		
Days off work	0·340 (0·340)	1·952 (0·758)	-1·613 (-3·357 to 0·019)

GP: general practitioner

5.2 Economic Costs

Table A4 summarises the total NHS and PSS costs associated with resource use during the trial period among complete cases, by cost category and follow-up period· The mean direct intervention costs were £35·71 for the Plaster Cast group compared with £108·64 for the Functional Brace group; the mean difference of £72·93 was statistically significant at the 5% level· The mean total NHS and PSS costs were significantly lower in the Functional Brace group between randomisation and 8-week post injury and between 8 weeks and 3 months post injury with mean between-group cost differences of £107·73 and £92·95, respectively· The mean total NHS and PSS cost throughout the entire follow-up period was £1182·64 for the Plaster Cast group and £1018·26 for the Functional Brace group; the mean between-group cost difference of £164·39 was not statistically significant at the 5% level·

Table A4: NHS and personal social services costs for cases with complete resource use data by trial allocation, study period and cost category (£, 2017-18 prices)

Cost category by period	Treatment group, mean (SE) Cost		Mean difference	p-value[a]	Bootstrap 95% CI[b]
	Plaster Cast	Functional Brace			
**Baseline to 8 weeks post injury – direct intervention costs[c] (total , n = 497: Plaster Cast group; n = 241; Functional Brace group, n =256)**					
Total direct intervention costs	35·71 (0·492)	108·64 (3·114)	-72·93	<0·0001	(-79·22 to -66·64)
**Baseline to 8 weeks post injury – NHS PSS resource use (total , *n* = 432: Plaster cast group; *n* = 210; Functional brace group, *n* =222)**					
Inpatient care	55·8 (28·382)	39·3 (22·163)	16·51	0·65	(-53·48 to 86·49)
Outpatient care	370·2 (15·114)	282·6 (15·078)	87·59	<0·0001	45·97 to 129·21)
Community care	9·66 (2·521)	28·94 (14·493)	-19·28	0·19	(-47·64 to 9·07)
Medications	151·35 (9·334)	106·45 (8·701)	44·9	<0·0001	20·34 to 69)
Aids and adaptations	9·51 (0·842)	7·32 (0·568)	2·19	0·032	(0·20 to 4·19)
Personal Social Services	0·15 (0·151)	0 (0)	0·15	0·32	(-0·14 to 0·45)
Total NHS and PSS cost	596·67 (36·596)	464·61 (32·946)	132·06	0·0080	(33·35 to 230·78)
Total Costs throughout first 8 weeks (including direct intervention costs)[d]	***647·88 (37·99)***	***540·15 (26·10)***	***107·73***	***0·02***	***(16·15 to 199·31)***
**8 weeks – 3months post injury (total , n = 434: Plaster Cast group; n = 207; Functional Brace group, n =227)**					
Inpatient care	61·69 (27·278)	4·74 (4·74)	56·95	0·041	(4·33 to 109·57)
Outpatient care	118·74 (9·948)	98·76 (7·608)	19·98	0·11	(-5·07 to 45·03)
Community care	31·96 (15·86)	19·89 (4·77)	12·07	0·34	(-20·69 to 44·83)
Medications	5·95 (3·359)	2·86 (2·133)	3·1	0·44	(-4·56 to 10·75)
Aids and adaptations	1·65 (0·359)	0·75 (0·261)	0·9	0·044	(0·014 to 1·78)

Cost category by period	Treatment group, mean (SE) Cost		Mean difference	p-value[a]	Bootstrap 95% CI[b]
	Plaster Cast	Functional Brace			
PSS	0 (0)	0·04 (0·04)	-0·04	0·32	(-0·12 to 0·039)
**Total NHS and PSS cost**	**220·00 (36·662)**	**127·04 (12·333)**	**92·95**	**0·017**	**(14·80 to 171·11)**
**3 – 6 months post injury (total , n = 436: Plaster Cast group; n = 210; Functional Brace group, n =226)**					
Inpatient care	21·08 (16·736)	43·94 (22·392)	-22·86	0·41	(-80·61 to 34·90)
Outpatient care	128·56 (11·731)	142·56 (13·215)	-14	0·43	(-47·66 to 19·67)
Community care	33·56 (2·617)	27·75 (5·679)	5·812	0·38	(-19·98 to 31·60)
Medications	0 (0)	1·51 (1·015)	-1·51	0·14	(-3·56 to 0·54)
Aids and adaptations	1·02 (0·472)	1·03 (0·534)	-0·01	0·99	(-1·38 to 1·36)
PSS	0·49 (0·491)	0 (0)	0·49	0·32	(-0·42 to 1·40)
**Total NHS and PSS cost**	**184·70 (26·350)**	**216·78 (29·988)**	**-32·07**	**0·42**	**(-108·74 to 44·58)**
**6 – 9 months post injury (total , n = 491: Plaster Cast group; n = 235; Functional Brace group, n =256)**					
Inpatient care	5·94 (4·771)	45·45 (45·45)	-39·51	0·39	(-133·06 to 54·05)
Outpatient care	76·44 (22·253)	65·98 (9·884)	10·46	0·67	(-39·34 to 60·26)
Community care	14·03 (4·022)	17·302 (7·197)	-3·27	0·69	(-19·78 to 13·24)
Medications	0·33 (0·228)	0·13 (0·058)	0·2	0·40	(-0·26 to 0·66)
Aids and adaptations	0·36 (0·217)	0·08 (0·052)	0·28	0·21	(-0·15 to 0·71)
PSS	0·11 (0·11)	0 (0)	0·11	0·32	(-0·10 to 0·32)
**Total NHS and PSS cost**	**97·21 (23·666)**	**128·94 (46·80)**	**-31·73**	**0·54**	**(-127·40 to 63·94)**
**0 – 9 months post injury (total , n = 292: Plaster Cast group; n = 134; Functional Brace group, n =158)**					
Total direct intervention costs	35·96 (0·646)	106·46 (4·08)	-70·5		(-78·30 to -62·69)
Inpatient care	162·29 (85·042)	45·43 (24·373)	116·86	0·19	(-62·66 to 296·38)
Outpatient care	722·78 (39·326)	653·42 (40·288)	69·36	0·22	(-38·61 to 177·33)
Community care	103·22 (33·32)	91·18 (22·809)	12·04	0·77	(-67·72 to 91·80)
Medications	146·41 (11·364)	112·61 (10·884)	33·8	0·033	(-2·66 to 64·94)
Aids and adaptations	11·75 (1·331)	9·16 (1·127)	2·59	0·14	(-0·71 to 5·88)
PSS	0·24 (0·236)	0 (0)	0·24	0·32	(-0·22 to 0·70)
**Total NHS and PSS costs throughout first 9 months**	**1182·64 (114·696)**	**1018·26 (58·143)**	**164·39**	**0·20**	**(-95·75 to 424·52)**

[a] *p*-value calculated using the student's t-test, two-tail unequal variance
[b] Non-parametric bootstrap estimation suing 1000 replications
[c] Time horizon for calculating total direct intervention costs was 8 weeks in order to capture costs associated with any changes required to either plaster cast or functional bracing (e·g· plaster cast changes)
[d] Total costs throughout first 8 weeks calculated based on total sample size of 415: Plaster cast, n=200; Functional brace, n=215) i·e· cases with complete intervention and resource use costs at 8 weeks·
PSS: Personal social services

5.3 Health outcomes

Table A5 summarised the EQ-5D Utility and EQ-5D VAS estimates and the estimated difference between the two treatment groups for each study period. The mean EQ-5D utility score was significantly lower at 8 weeks post injury in the Functional Brace group (0·588 versus 0·655; p<0·0001) amongst complete cases· However, there were no statistically significant differences in EQ-5D utility scores between the treatment groups at any other follow-up time-point· There were no statistically significant differences in the Visual Analogue Scale scores between the comparator groups·

Table A5: EQ-5D Utility and EQ-5D VAS mixed effects model results at 8, 3, 6 and 9 months post injury (modified ITT population) by trial allocation and study period

	Plaster Cast (n = 266)		Functional Brace (n = 274)		Between - Group Difference (95% CI)		
	Mean (SD)	n	Mean (SD)	n	Unadjusted	Adjusted[b]	p-value
**EQ-5D Utility**							
Baseline post injury	0·242 (0·02, ·47)[a]	264	0·282 (0·03, 0·52)[a]	273	0·042 (0·01, 0·08)	0·041 (0·01, 0·07)	0·017
8 week	0·588 (0·23)	234	0·655 (0·18)	241	0·066 (0·03, 0·1)	0·069 (0·03, 0·1)	0·00051
3 month	0·638 (0·22)	229	0·669 (0·19)	245	0·031 (-0·01, 0·07)	0·035 (0, 0·07)	0·056
6 month	0·766 (0·15)	224	0·757 (0·18)	237	-0·009 (-0·05, 0·03)	-0·002 (-0·04, 0·03)	0·916
9 month	0·829 (0·72, 0·91)[a]	244	0·795 (0·72, 0·88)[a]	259	-0·010 (-0·05, 0·03)	-0·009 (-0·04, 0·03)	0·623
**EQ-5D VAS**							
Baseline post injury	90·0 (80, 95)[a]	263	90·0 (80, 95)[a]	273	0·77 (-2·18, 3·72)	1·28 (-1·4, 3·97)	0·349
8 week	75·0 (60, 85)[a]	234	75·0 (65, 85)[a]	240	1·08 (-2·05, 4·2)	1·61 (-1·21, 4·43)	0·264
3 month	80·0 (65, 85)[a]	229	80·0 (65, 90)[a]	245	1·29 (-1·84, 4·42)	1·66 (-1·16, 4·48)	0·249
6 month	81·5 (70, 90)[a]	224	80·0 (70, 90)[a]	236	0·49 (-2·69, 3·66)	1·08 (-1·77, 3·93)	0·458
9 month	86·0 (80, 92)[a]	242	85·0 (75, 91)[a]	259	-0·76 (-3·8, 2·28)	-0·56 (-3·32, 2·2)	0·693

[a] Median (Interquartile Range); [b] Analysis adjusted for site, age, gender and EQ-5D baseline pre-injury with repeated observations within participant

VAS: Visual Analogue Scale; CI: Confidence Interval; SD: standard deviation

5.4 Cost-effectiveness results

The cost-effectiveness results are presented in Table A6~~5~~ with Plaster Cast selected as the referent and Functional Brace as the comparator, i·e· functional brace minus plaster cast, for the estimation of ICER values· The analytic time horizon covers the entire 9-month post-injury follow-up period of the trial· The joint distribution of costs and outcomes for the base-case analysis is graphically represented in Figure A1·

5.4.1 Base case analysis

Patients in the Functional Brace group experienced a non-statistically significant increase in QALYs in the base case (0·015 QALYs, 95% CI: -0·0013 to 0·030) over the 9-month follow-up period· Mean NHS and PSS costs were also lower in the Functional Brace group [mean cost difference: -£103 (95% CI: -289 to 84)]· The ICER for the base-case analysis indicates that functional bracing is the dominant procedure as average costs for this intervention were lower whilst average benefits were greater than those for plaster cast·
Assuming cost-effectiveness thresholds of £15000 per QALY, £20000 per QALY and £30000 per QALY, respectively, the probability of cost-effectiveness for functional bracing ranged from 0·96 to 0·97, whilst the NMB associated with functional bracing was positive (Table A~~5~~6; Figure A2)·

5.4.2 Sensitivity analyses

Comparing mean costs and QALY estimates using different analytical scenarios (complete case, societal perspective and CACE population) revealed that the cost-effectiveness results generally supported the base case finding, with the exception of the sensitivity analysis that adopted a societal perspective· For the societal

perspective, mean costs were higher in the Functional Brace group (£248, 95% CI: -476 to 972)· However, the QALY results followed the same pattern as that for the base case analysis and indicated that participants in the Functional Brace group experienced a non-statistically significant increase in QALYs over the 9-month follow-up period (0·015 QALYs, 95%CI: -0·0042 to 0·031)· The probability of cost-effectiveness of functional brace declined to a range of 0·50 to 0·69 at cost-effectiveness thresholds of £15000 per QALY, £20000 per QALY and £30000 per QALY· The results of the mixed effects model followed a similar pattern to that of the base case (imputed) model: Patients in the Functional Brace group experienced a non-statistically significant increase in QALYs (0·014 QALYs, 95% CI: -0·0018 to 0·031) over the 9-month follow-up period· Mean NHS and PSS costs were also lower in the Functional Brace group [mean cost difference: -£135 (95% CI: -342 to 71)]·

5.4.3 Long-term economic modelling

The protocol allowed for decision-analytic modelling to estimate the longer-term cost-effectiveness of functional bracing or plaster cast· However, we note that cost and health utility values started to converge from the 3-month follow-up time-point and converged at subsequent time points, even though functional brace was cost-effective over the entire follow-up period· It was therefore concluded that longer-term extrapolation of cost-effectiveness of functional is highly unlikely to be meaningful· Furthermore, we did not identify external studies that compared differences in economic costs, functional outcomes or health-related quality of life beyond 9 months post injury in non-surgical patients treated with a plaster cast or functional brace· This lack of data needed to parameterize a model further challenged any efforts to conduct longer-term decision modelling·

Table A65: Cost-effectiveness, cost/QALY (£, 2017): functional brace compared to plaster cast

	Treatment group, mean (SE) Cost		Incremental cost (95% CI)	Treatment group, mean (SE) QALY		Incremental QALYs (95% CI)	ICER*	Probability of cost-effectiveness			Net monetary benefits		
**Scenario**	**Functional Brace**	**Plaster Cast**		**Functional Brace**	**Plaster Cast**			**P^1**	**P^2**	**P^3**	**NMB[1] (95% CI[6])**	**NMB[2] (95% CI[6])**	**NMB[3](95% CI[6])**
**Base case analysis**													
Imputed attributable costs and QALYs, covariate adjusted	1078·16 (83·42)	1180·72 (89·63)	-102·56 (-289·28 to 84·16)	0·506 (0·0064)	0·492 (0·0066)	0·015 (-0·0013 to 0·030)	Dominant	0·963	0·965	0·966	312·28 (-31·26 to 655)	383·82 (-32·67 to 793·80)	526·90 (-42·50 to 1076·87)
**Sensitivity analyses**													
Complete case attributable costs and QALYs, covariate adjusted	948·77 (53·91)	1117·28 (110·66)	-168·51 (-458·01 to 32·88)	0·513 (0·00642)	0·497 (0·0064)	0·017 (-0·0035 to 0·037)	Dominant	0·976	0·976	0·972	443·54 (19·83 to 933·22)	527·26 (9·11 to 1094·07)	694·70 (-17·56 to 1406·23)
Societal perspective	4362·15 (348·71)	4114·54 (292·18)	247·61 (-476·44 to 971·66)	0·506 (0·0063)	0·502 (0·007)	0·015 (-0·0042 to 0·031)	16510	0·501	0·576	0·688	-29·65 (-991·50 to 874·93)	44·36 (-964·19 to 991·46)	192·39 (-926·97 to 1244·53)
CACE[4] population	1038·6 (62·89)	1169·44 (78·48)	-130·84 (-335·38 to 90·36)	0·510 (0·00609)	0·488 (0·00688)	0·022 (0·0051 to 0·038)	Dominant	0·992	0·993	0·994	44·52 (89·86 to 852·63)	57·36 (127·50 to 1030·39)	818·02 (199·26 to 1434·03)
Secondary cost-effectiveness analysis using ATRS[4] as outcome measure	1057·22 (71·91)	1149·44 (79·25)	-92·21 (-273·86 to 89·44)	45·09 (0·72)	44·30 (0·73)	0·78 (-1·12 to 2·69)	Dominant	0·875	0·839	0·822	174·03 (-117·37 to 463·44)	328·84 (-306 to 970·91)	406·25 (-403·75 to 1218·76)

*ICER: Incremental cost-effectiveness ratio· Given the pattern of results, Plaster Cast has been selected as the referent and Functional Brace as the comparator, i·e· functional brace minus plaster cast, for the estimation of ICER values· Dominance indicates average costs were less and average benefit greater for functional brace vs· plaster cast

P^1, P^2, P^3: probability cost-effective if cost-effectiveness threshold set at £15,000/QALY, £20,000/QALY or £30,000/QALY, respectively with the exception of the sensitivity analysis using ATRS as the health outcome measure of interest· In the latter case P^1, P^2, P^3 refer to probability of cost-effectiveness if cost-effectiveness threshold arbitrarily set at £100; £300 and £500 per unit gain in ATRS score

NMB[1], NMB[2],NMB[3]: net monetary benefit if cost-effectiveness threshold set at £15,000/QALY, £20,000/QALY or £30,000/QALY, respectively with the exception of the sensitivity analysis using ATRS as the health outcome measure of interest· In the latter case NMB[1], NMB[2],NMB[3] refer to net monetary benefit if cost-effectiveness threshold arbitrarily set at £100; £300 and £500 per unit gain in ATRS score

[4] CACE Complier Average Causal Effect

[5] ATRS (Achilles Tendon Rupture Score) range from 0 to 100 with higher scores indicating better outcome

[6] CI: confidence interval

QALY: quality adjusted life-year

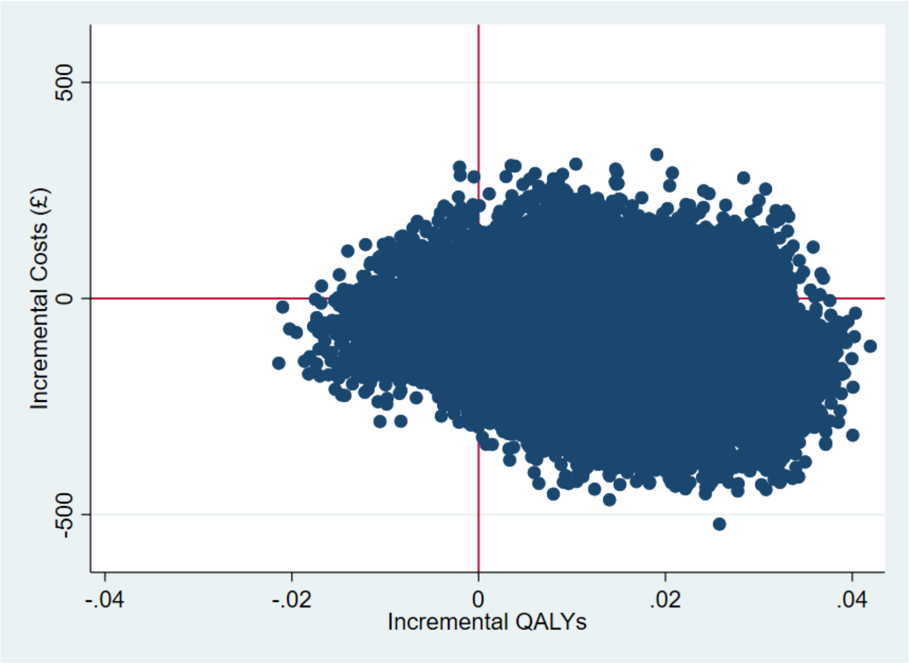

Figure A1: Cost-effectiveness scatterplot at 9 months for base case analysis (NHS and personal social service perspective, imputed- additionally controlled for pre-injury utility, intention-to-treat analysis)

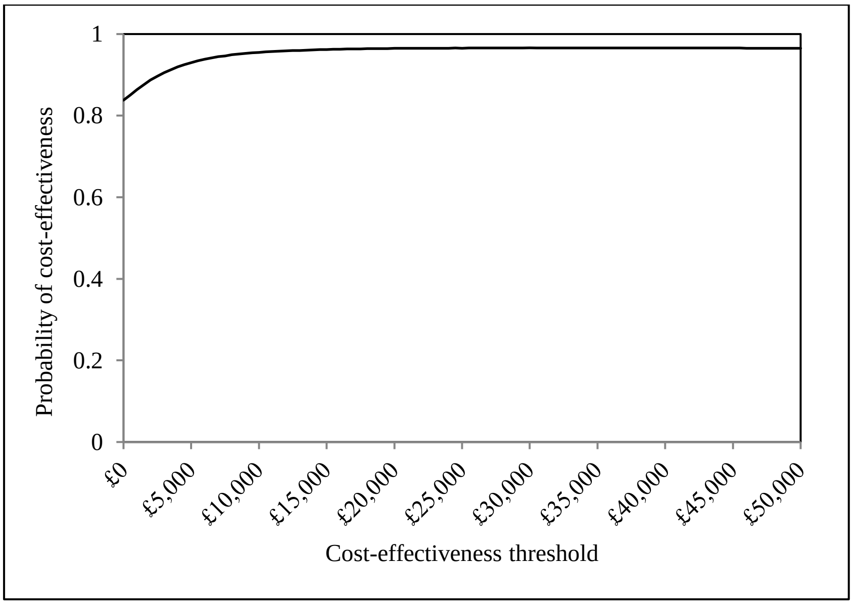

Figure A2: Cost-effectiveness acceptability curve at 9 months for base case analysis (NHS and personal social service perspective, imputed- additionally controlled for pre-injury utility, intention-to-treat analysis)

References appendix 3

1· National Institute for Health and Care Excellence (NICE)· Guide to the methods of technology appraisal· *National Institute for Health and Care Excellence (NICE)* 2013·

2· Curtis LA, Burns, A· Inflation indices· In: Unit Costs of Health and Social Care 2018· Kent: Personal Social Services Research Unit; 2018: 166·

3· Curtis LA, Burns, A· *Unit costs of health and social care 2018* no· 1902671619· Canterbury: University of Kent; 2018·

4· NHS Improvement· *Reference Costs 2017-2018*· Department of Health, Editor· London; 2018·

5· NHS Digital· *Prescription Cost Analysis - England, 2017*· Department of Health, Editor· London: NHS Digital; 2018·

6· Joint Formulary Committee· *British National Formulary*· London: BMJ Group and Pharmaceutical Press 2018· URL: https://www·evidence·nhs·uk/formulary/bnf/current (Accessed 12 August, 2016)·

7· NHS· *NHS Supply Chain*· 2018· URL: https://my·supplychain·nhs·uk/catalogue (Accessed 18 July, 2018)·

8· Office for National Statistics· *Employee earnings in the UK: 2018*· Office for National Statistics, Editor· London; 2018·

9· Herdman M, Gudex C, Lloyd A, Janssen M, Kind P, Parkin D, et al· Development and preliminary testing of the new five-level version of EQ-5D (EQ-5D-5L)· *Quality of Life Research* 2011 Dec;20(10):1727–36·

10· van Hout B, Janssen MF, Feng Y-S, Kohlmann T, Busschbach J, Golicki D*, et al*· Interim Scoring for the EQ-5D-5L: Mapping the EQ-5D-5L to EQ-5D-3L Value Sets· *Value in Health* 2012;**15**:708-15· http://dx·doi·org/https://doi·org/10·1016/j·jval·2012·02·008

11· Faria R, Gomes M, Epstein D, White IR· A guide to handling missing data in cost effectiveness analysis conducted within randomised controlled trials· *PharmacoEconomics* 2014; 32(12): 1157-70·

12· Rubin DB: Multiple imputation for nonresponse in surveys· John Wiley & Sons, 2004·

13· Claxton K, Martin S, Soares M, Rice N, Spackman E, Hinde S*, et al*· Methods for the estimation of the National Institute for Health and Care Excellence cost-effectiveness threshold· *Health technology assessment (Winchester, England)* 2015;**19**:1-503· http://dx·doi·org/10·3310/hta19140

UKSTAR trial collaborators

Ines Reichert, King's College Hospital;
Andrew McAndrew, Royal Berkshire Hospital;
Alan Johnstone, Aberdeen Royal Infirmary;
Fraser Harrold, NHS Tayside – Dundee;
Jane Madeley, Glasgow Royal Infirmary;
Harish Kurup, Pilgrim Hospital, Boston, United Lincolnshire Hospitals NHS Trust;
Rajesh Nanda, University Hospital of North Tees;
Avijeet Ghosh, Airedale General Hospital;
Sridhar Sampalli, Salisbury District Hospital;
Sandeep Kapoor, Rotherham Hospital;
Asterios Dramis, George Eliot Hospital NHS Trust;
Nitin Modi, James Paget University Hospital NHS Foundation Trust;
Nicholas Hancock, University Hospital Southampton NHS Foundation Trust;
Rupe Deol, Lister Hospital, East and North Herts NHS Trust;
Richard Walter, Royal Cornwall Hospital;
Justin Forder, Maidstone and Tunbridge Wells NHS Trust;
Peter Hull, Cambridge University Hospitals – Addenbrookes;
Mark Westwood, Derriford Hospital, Plymouth;
Viren Mishra, Hull Royal Infirmary;
Simon Burtt, Luton and Dunstable Hospital;
Victoria Lyle, Salford Royal NHS Foundation Trust;
Nikos Reissis, Northern Lincolnshire & Goole;
Jason Eyre, Mid Yorkshire NHS Trust;
Paul Harwood, Leeds General Infirmary;
Abhijit Guha, Alexandra Hospital Redditch;
Sanjeev Madan, Doncaster and Bassetlaw Hospitals NHS Foundation Trust;
Andrea Sott, Epsom and St Helier University Hospitals NHS Trust;
Rajarshi Bhattacharya, Imperial College Healthcare NHS Trust;
James Beastall, Raigmore Hospital Inverness;
Jordi Ballester, Whiston Hospital;
Atif Malik, Milton Keynes University Hospital;
Sameh El-Kawy, Warwick Hospital;
Babis Karagkevrekis, Burton Hospitals NHS Foundation Trust;
Amr Abdallah, Hereford County Hospital;
Ansar Mahmood, Queen Elizabeth Hospital Birmingham;
Mark Deakin, John Radcliffe Oxford University Hospitals;
Moez Ballal, Nasser Kurdy, South Manchester NHS Foundation Trust
Andrew Kelly, Musgrove Park Hospital Taunton.
